# Comparative effectiveness of pharmacological treatments for patients with diarrhea-predominant irritable bowel syndrome: Protocol of a systematic review and network meta-analysis

**DOI:** 10.1097/MD.0000000000011682

**Published:** 2018-08-03

**Authors:** Ling Yue, Min Chen, Tai-Chun Tang, Tian-Wei She, Yao-Yao Chen, Hui Zheng

**Affiliations:** a Acupuncture and Tuina School/3rd Teaching Hospital; b School of Clinical Medicine/Teaching Hospital, Chengdu University of Traditional Chinese Medicine, Chengdu, Sichuan, China.

**Keywords:** diarrhea-predominant irritable bowel syndrome, harmacological treatments, network meta-analysis, study protocol

## Abstract

**Background:**

Several pharmacological treatments are recommended by guidelines with moderate to high evidence for the treatment of diarrhea-predominant irritable bowel syndrome (IBS-D), but the comparative effectiveness and safety among these treatments are unknown. The review is to assess the comparative effectiveness and safety of pharmacological treatments for IBS-D using network meta-analysis.

**Methods:**

We will search Ovid Medline, EMBASE, and the Cochrane Central Register of Controlled Trials (CENTRAL) for relevant randomized controlled trials (RCTs) that compare guideline-recommended pharmacological treatments with placebo or one of the treatments. We will include RCTs that recruit patients with IBS-D, RCTs that assess the improvement in IBS-D global symptoms, abdominal pain, stool frequency, or stool consistency, and RCTs that assess the responder rate and adverse event rate. We will use standardized mean difference to synthesize continuous variables and use odds ratio to synthesize categorical variables. Traditional meta-analysis will be performed to assess the comparative effectiveness of the pharmacological treatments in direct evidence, and network meta-analysis will be performed to combine both direct and indirect evidence. Transitivity of the evidence in the network will be assessed by using a generalized Cochrane *Q* statistic and net-heat plot.

**Conclusions:**

The result of the review will inform clinical decisions for clinicians, patients, and police makers in the treatment of IBS-D.

**Results:**

Ethical approval and informed consent are not required for this systematic review. We will disseminate the result through a peer-reviewed journal and conference abstracts.

**PROSPERO registration number:**

PROSPERO CDR42018099294.

## 1 Introduction

Irritable bowel syndrome (IBS) is a functional bowel disorder characterized by abdominal pain or discomfort that are correlated to bowel movements or changes in bowel habits.^[1]^ The prevalence of IBS in the general population is approximately 10% to 25%.^[2]^ IBS with diarrhea (IBS-D) accounts for about 40% of IBS.^[3]^ IBS-D decreases quality of life, and it raises indirect health-care cost and causes heavy social burden.^[4]^

The etiology and pathogenesis of IBS-D are not fully understood. It is closely correlated to visceral hypersensitivity, dysfunctions in colonic motility, disorder in serotonin secretion, abnormal gut flora, and psychological disorders.^[5,6]^ The treatment focuses mainly on relieving symptoms and improving the quality of life.^[7]^ The guidelines recommend patients with IBS-D to use antispasmodic agents as their first choice; when they fail to improve IBS-D symptoms, antidepressants could be selected.^[8]^ However, the antispasmodic agents have low quality evidence in their effectiveness due to clinical trial design problems, small sample size, and other reasons^[9,10]^; antidepressants are often used for patients with moderate to severe IBS-D, and their generalizability in clinical practice is limited because of their intolerability and lack of regular follow-up.^[8,10,11]^ Recently, new drugs acting on serotonin and opioid system are developed for the purpose of adding new options for patients with IBS-D.^[12]^ 5-HT_3_ receptor antagonists (e.g., alosetron) slow down bowel transmission, enhance intestinal fluid reabsorption, and reduce IBS-related visceral pain,^[13]^ but they have side effects causing constipation and ischemic colitis. The Food and Drug Administration (FDA) recommend using alosetron only for women with IBS-D who have severe IBS-D and fail to respond to conventional treatments^[14]^; alosetron is reported to be superior over antispasmodic agents in the treatment of women with non-constipated IBS.^[15]^ Eluxadoline, one of the opioid receptor ligands, has shown significant effect on improving abdominal pain, fecal frequency, and urgency to bowel movements in patients with IBS-D in 2 phase-III trials.^[16]^ Rifaximin is approved for improving IBS global symptoms and abdominal distension in patients with IBS-D by the FDA^[17,18]^; it shows good safety and tolerability along with its effectiveness.^[19]^ Based on these grounds, we raise a clinically important question: which of these pharmacological treatments is the most comparatively effective and safe? To help patients and clinicians make better choice in treating IBS-D, we will conduct a systematic review and network meta-analysis to compare the efficacy and safety of these drugs combining both direct and indirect evidence.

## 2 Methods

### 2.1 Design and registration of the review

This systematic review and network meta-analysis will assess the comparative effectiveness and safety of pharmacological treatments for IBS-D. The protocol of the review conforms to the Preferred Reporting Items for Systematic review and Meta-analysis Protocols (PRISMA-P),^[20]^ and it has been registered in PROSPERO (https://www.crd.york.ac.uk/PROSPERO/, CRD).

### 2.2 Study source

We will search OVID MEDLINE, EMBASE, and the Cochrane Central Register of Controlled Trials (CENTRAL) for randomized controlled trials (RCTs) testing the efficacy of guideline-recommended treatments or treatments with moderate to high evidence in the management of IBS-D. We develop a comprehensive search strategy using keywords and Mesh terms in combination to search for target RCTs; the search strategy sample is provided in Table 1. We will also search clinical registries (clinicaltrials.gov, eudract.ema.europa.eu, and www.isrctn.com) for ongoing RCTs, and we will contact the investigators of these trials to ask for preliminary data if possible. Systematic reviews examining the effect of 5-HT_3_ receptor antagonists, opioid receptor ligands, antidepressants, antibiotics on IBS-D will be retrieved, and we will screen the reference of the systematic reviews to search for relevant RCTs.

**Table 1:**
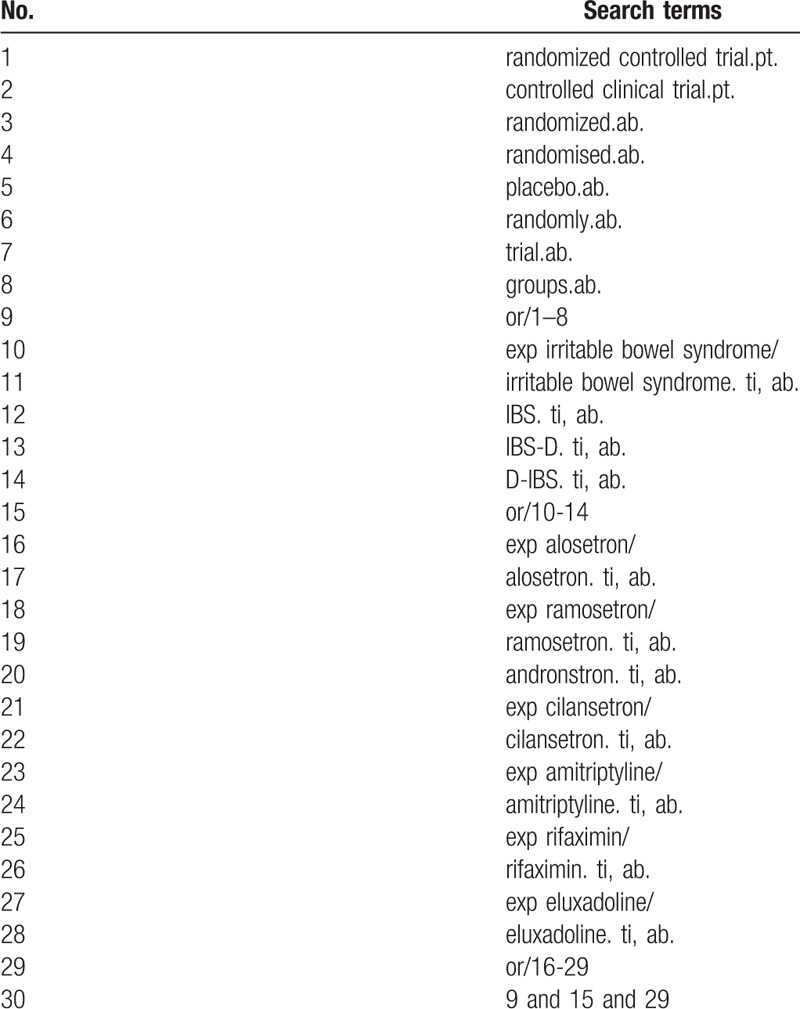
Search strategy.

### 2.3 Study design

We will include RCTs with parallel design, which compare the pharmacological treatments with placebo or one of these pharmacological treatments. RCTs with crossover design or N-of-1 design will be excluded.

### 2.4 Participants

We will include adult patients with IBS-D according to Rome I, II, III, or IV criteria and exclude those accompanied with inflammatory bowel diseases, gastrointestinal tumor, severe depressive symptoms, or hemafecia. We will include participants meeting at least one of the following conditions: with a mean score of visual analog scale (VAS) >3 cm in the assessment of global IBS-D symptoms, abdominal pain, abdominal distension, or defecation urgency (a VAS score ranges from 0 to 10 cm, with 0 indicating no symptoms and 10 indicating the worst symptom); with a Bristol score of 6 or 7 (a Bristol score ranges from 1 to 7; a score of 6 or 7 indicates diarrhea).

#### 2.4.1 Interventions and comparisons

Pharmacological treatments recommended by the guideline^[8]^ or treatments with moderate to high evidence (defined as having at least 2 phase III RCTs showing the effectiveness of a treatment) will be included. These treatments are: 5-HT_3_ receptor antagonists (alosetron, ramosetron, andronstron, and cilansetron), antidepressants (amitriptyline), antibiotics (rifaximin), and opioid receptor ligands (asimadolin, eluxadoline). The treatments should be used for at least 2 weeks. RCTs using flexible doses or fixed dose of a treatment will be included. The fixed dose of a treatment should be the minimally effective dose recommended by the aforementioned guidelines. We will include RCTs comparing these treatments with placebo or one of these treatments. We will include treatments used as monotherapy, or on the basis of dietary interventions (e.g., low FODMAP diet),^[21]^ or in combination with other guideline-recommended treatments. We also include treatments used for treating the relapse of IBS-D symptoms (e.g., rifaximin is used after 5-HT_3_ receptor antagonists fail).

### 2.5 Outcome measurements

The primary outcome will be the improvement of global symptoms assessed with VAS. The secondary outcomes include the improvement of major IBS-D symptoms assessed with VAS (e.g., abdominal pain, bloating, and defecation urgency), the improvement of the mean stool frequency per week, the improvement of stool consistency assessed with the Bristol score,^[22]^ responder rate (a responder is defined as a participant having at least 30% reduction in the VAS score of IBS-D global symptoms, abdominal pain, or stool frequency), and adverse event rate.

### 2.6 Study screening and data extraction

Two reviewers (LY and TWS) will independently screen the titles and abstracts of retrieved studies. If the 2 reviewers cannot determine whether a study should be included according to its title or abstract, they will further examine the full-text of the study. Another 2 reviewers (YYC and TCT) will read the full-text of the included studies and extract information from them with standardized data extraction forms. They will extract the information of trial characteristics, participants’ characteristics, interventions, and outcome assessments. The trial characteristics will include study title, clinical registry number, the year of publication, dataset (intention-to-treat, per-protocol, or as-treated), study design (double blind, single blind, or open label), study duration, and total sample size. The participants’ characteristics will include age, sex, duration of IBS-D, baseline VAS score of IBS-D symptoms (global symptoms, abdominal pain, bloating, and defecation urgency), refractory IBS-D (defined as treatment failure after at least one of the guideline-recommended treatments), and accompanied conditions. Intervention characteristics will include the name of intervention, the number of participants receiving experimental interventions or controls, dosage, treatment duration, and accompanied treatments. Outcome assessments will include the assessment time points and the values of the outcomes. YYC and TCT will contact the authors for missing information in the articles.

### 2.7 Risk of bias assessment

We will assess the risk of bias with a tool recommended by the Cochrane collaboration.^[23]^ We will evaluate the risk of bias in generating random sequence, random sequence concealment, blinding, incomplete data reporting, selective outcome reporting, and other risk, and we will classify RCTs having low risk of bias in the first 3 items as high-quality RCTs.

### 2.8 Data synthesis

We will qualitatively summarize the included RCTs, describing the characteristics of study design, participants, interventions, outcome measures, and main outcomes. Missing values that cannot be acquired from the authors of the RCTs will be handled in reference to the Cochrane handbook.^[23]^ When an outcome is assessed at multiple time points in a RCT, we will combine the outcome assessments in all time points with a multivariate meta-analysis model.^[24]^ We will firstly group interventions in each category (e.g., we will group rifaximin in antibiotics) and calculate the effect sizes and related 95% confidence intervals (CIs) of each category via conventional pairwise meta-analysis, and we secondly repeat the calculation again for each individual intervention used in different doses (e.g., rifaximin used at a dose of 550 mg). We will calculate the effect sizes of continuous data with standardized mean difference (SMD), and we will calculate the effect sizes of categorical data with odds ratio (OR). SMDs are recognized as small, median, and large effect size by using 0.2, 0.5, and 0.8 as cut-off points, respectively.^[25]^ RCTs containing a treatment with zero event will be excluded from the meta-analysis.

We will perform network meta-analysis combining direct and indirect comparisons from the included RCTs. The network meta-analysis will be performed within a frequentist framework and calculated by using the *netmeta* package in R software (www.r-project.org, version 3.2.0). Network geometry will be examined by a network plot showing the number of participants assigned to each treatment and the number of direct comparisons made between 2 treatments. We will rank the treatments on the basis of *P* scores of the included treatments. The *P* score measures the extent of certainty that a treatment is superior to another treatment without the need to use resampling method.^[26]^

Transitivity of the network meta-analysis will be examined by comparing the result of direct comparisons with indirect comparisons, and a Z test will be used to examine whether significant difference exists between them. We will measure within-design and between-design heterogeneity by using a designed-based decomposition of Cochran *Q*.^[27]^ We will use a net-heat plot to show the disagreement between different source of evidence comparing any 2 of the treatments and the contribution of each source of the evidence to the effect estimate. When significant heterogeneity is found, we will perform meta-regression to determine the source of heterogeneity. The source to be examined in the meta-regression include the duration of IBS-D, baseline VAS score of IBS-D symptoms, refractory IBS-D, and the year of publication (before 2000, 2001–2010, and 2011–2018).

We will run subgroup analyses. Firstly, we will separately reperform the meta-analysis in participants receiving oral administration of the treatments and those using other administration methods. Secondly, we will separately analyze RCTs with study period under 3 months and those >3 months. We will perform sensitivity analyses. Firstly, we will exclude low-quality RCTs (defined as having high or unclear risk of bias in random sequence generation, random sequence concealment, and blinding) and re-run the meta-analysis. Secondly, we will exclude RCTs with the number of participants <100 per group to control the small study effect. Thirdly, we will exclude RCTs using the PP dataset.

## 3 Discussion

Recently, tons of RCTs have tested pharmacological treatments that are reported specifically for patients with IBS-D. So we raise questions on which treatment has the best treatment effect and the least harms. The study protocol of this network meta-analysis is therefore conceived and designed. The result of this meta-analysis will add knowledge in the comparative effectiveness and safety of current pharmacological treatments, which helps patients and physicians make the most suitable decisions on their individualized selection for treatment. It also helps health policymakers to develop recommendations according to their own socioeconomic situations.

## Author contributions

**Conceptualization:** Ling Yue, Min Chen, Hui Zheng.

**Data curation:** Tian-Wei She, Yao-Yao Chen.

**Formal analysis:** Min Chen, Hui Zheng.

**Investigation:** Ling Yue, Tai-Chun Tang, Tian-Wei She, Yao-Yao Chen.

**Methodology:** Ling Yue, Min Chen, Hui Zheng.

**Writing – original draft:** Ling Yue, Min Chen, Hui Zheng.

**Writing – review and editing:** Ling Yue, Min Chen, Hui Zheng.

**Investigation:** Ling Yue, Min Chen, Tai-Chun Tang, Tian-Wei She, Yao-Yao Chen.

**Writing – review and editing:** Hui Zheng.
